# Defensive alteration of root exudate composition by grafting *Prunus* sp. onto resistant rootstock contributes to reducing crown gall disease

**DOI:** 10.1093/hr/uhae049

**Published:** 2024-02-23

**Authors:** Lin Chen, Lusen Bian, Qinghua Ma, Ying Li, Xinghong Wang, Yunpeng Liu

**Affiliations:** National Permanent Scientific Research Base for Warm Temperate Zone Forestry of Jiulong Mountain, Experimental Center of Forestry in North China, Chinese Academy of Forestry, Beijing 102300, China; National Permanent Scientific Research Base for Warm Temperate Zone Forestry of Jiulong Mountain, Experimental Center of Forestry in North China, Chinese Academy of Forestry, Beijing 102300, China; National Permanent Scientific Research Base for Warm Temperate Zone Forestry of Jiulong Mountain, Experimental Center of Forestry in North China, Chinese Academy of Forestry, Beijing 102300, China; National Permanent Scientific Research Base for Warm Temperate Zone Forestry of Jiulong Mountain, Experimental Center of Forestry in North China, Chinese Academy of Forestry, Beijing 102300, China; National Permanent Scientific Research Base for Warm Temperate Zone Forestry of Jiulong Mountain, Experimental Center of Forestry in North China, Chinese Academy of Forestry, Beijing 102300, China; State Key Laboratory of Efficient Utilization of Arid and Semi-arid Arable Land in Northern China, Institute of Agricultural Resources and Regional Planning, Chinese Academy of Agricultural Sciences, Beijing 100081, China

## Abstract

Grafting is a traditional and significant strategy to suppress soil-borne diseases, such as the crown gall disease caused by tumorigenic *Agrobacterium* and *Rhizobium*. Root exudates and the rhizosphere microbiome play critical roles in controlling crown gall disease, but their roles in suppressing crown gall disease in grafted plants remain unclear. Here, disease-susceptible cherry rootstock ‘Gisela 6’ and disease-resistant cherry rootstock ‘Haiying 1’ were grafted onto each other or self-grafted. The effect of their root exudates on the soil microbiome composition and the abundance of pathogenic *Agrobacterium* were studied. Grafting onto the disease-resistant rootstock helped to reduce the abundance of pathogenic *Agrobacterium*, accompanied by altering root exudation, enriching potential beneficial bacteria, and changing soil function. Then, the composition of the root exudates from grafted plants was analyzed and the potential compounds responsible for decreasing pathogenic *Agrobacterium* abundance were identified. Based on quantitative measurement of the concentrations of the compounds and testing the impacts of supplied pure chemicals on abundance and chemotaxis of pathogenic *Agrobacterium* and potential beneficial bacteria, the decreased valine in root exudates of the plant grafted onto resistant rootstock was found to contribute to decreasing *Agrobacterium* abundance, enriching some potential beneficial bacteria and suppressing crown gall disease. This study provides insights into the mechanism whereby grafted plants suppress soil-borne disease.

## Introduction

Soil-borne crown gall disease caused by tumorigenic agrobacteria affects a broad range of plant species worldwide. Crown gall disease always results in destructive production reduction in plants [1]. Tumor-inducing (Ti) plasmids are indispensable for the pathogenicity of agrobacteria [2]. Most species of tumorigenic strains belong to *Agrobacterium*; in addition, *Allorhizobium vitis* (formerly *Agrobacterium vitis*) and *Rhizobium rhizogenes* (formerly *Agrobacterium rhizogenes*) also cause crown gall disease [3–5].

Many strategies have been developed and applied to control crown gall disease, such as grafting, transgenic approaches, chemical agents, and biocontrol by beneficial bacteria [6–8]. Among these strategies, grafting is an effective method to suppress soil-borne pathogens, and has been used to resist diseases such as the insect pathogen phylloxera, fusarium wilt, and crown gall disease [9–11]. Besides improving plant disease resistance, grafting was also used to increase yields, allow plants to grow in new environments, and propagate desirable varieties of fruits, since many cultivated trees are not true-breeding (their seeds will not produce fruit identical to the parents) [10]. In conclusion, grafting is a widely used technique in agriculture and horticulture that is important for orchards, greenhouses, vineyards, and garden industries [10].

The composition of root exudates of grafted plants can be altered by metabolites of different scions or rootstocks [12]. Root exudates play a critical role in shaping the soil microbiome and regulating the abundance of soil-borne pathogens. Root exudates could favor, inhibit, or terminate microbial activity and growth [13, 14]. Root exudates include low molecular weight compounds and high molecular weight compounds [15, 16], the former being more diverse and including amino acids, sugars, organic acids, phenolics, and other secondary metabolites [17]. Some compounds of root exudates could help plants resist pathogen invasion. For instance, benzoxazinoids from maize root exudates could defend against pathogenic attacks by regulating innate immunity in maize [18]. The reduced secretion of raffinose from cucumber roots contributes to decreasing root colonization by *Fusarium oxysporum* f. sp. *cucumerinum* [19]. The alteration in the composition of defensive root exudates has a profound effect on pathogen abundance [17]. Ling *et al*. and Song *et al*. [20, 21] demonstrated that the alteration of chlorogenic acid, caffeic acid and some disease resistance proteins in root exudates might contribute to the ability of grafted watermelon to fight against *F. oxysporum* f. sp. *niveum*.

The composition of root exudates varies substantially among different plant species and cultivars, as do their rhizomicrobiome and plant–soil feedback effects [22, 23]. Root exudation can drive plant–soil feedbacks through assembling soil microbiota [24]. There is increasing evidence that plants recruit beneficial microbes in the rhizosphere to fight against disease by releasing root exudates [25, 26]. For example, some organic acids in root exudates contribute to recruiting beneficial *Bacillus* strains to inhibit pathogen growth [27, 28]. The disease-resistant tomato cultivar Hawaii 7996 can recruit flavobacterium TRM1 to protect plants from pathogenic *Ralstonia solanacearum* [23]. In addition, reducing the soil-borne pathogen ‘helpers’ in the microbiome composition has also been proposed to be a strategy to suppress disease [29].

**Figure 1:**
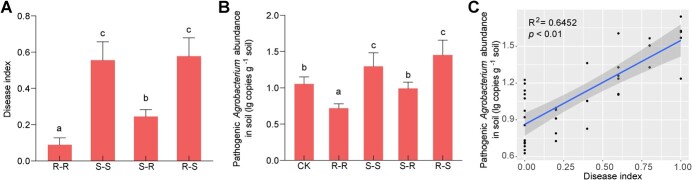
Effect of different grafted plants on pathogenic *Agrobacterium* abundance and crown gall disease index. **A** Disease index of different grafted plants was determined after plants were inoculated with pathogenic *Agrobacterium* for 60 days. **B** Quantification of pathogenic *Agrobacterium* in soil treated with root exudates of different grafted plants. Two hundred grams of soil was treated with 100 ml of the original concentration of root exudates of different grafted plants every week. Sterile water was used as a control. The soil was treated with root exudates for 6 weeks. **C** Pearson correlation between disease index and pathogen abundance. Values are mean ± standard deviation of nine replicates. Different letters above the columns indicate statistically significant differences between treatments (*P* < 0.05).

Grafting is an ancient technology to control soil-borne crown gall disease, and several mechanisms in grafted plants have been well studied and proposed [30–32]. However, the effect of grafting on root exudates and the mechanism by which grafted plants confer plant resistance to soil-borne crown gall disease by root exudation is still unclear. We hypothesize that the root exudates of a plant grafted on resistant cherry rootstock would reduce crown gall disease. In this study we used cherry cultivar (*Prunus* sp.) ‘Haiying 1’, which is highly resistant to crown gall disease, and ‘Gisela 6’, a cherry cultivar that is susceptible to crown gall disease, for grafting. We first assessed the changes in abundance of pathogen, composition of root exudates, and soil microbiota in different grafted plants and analyzed the link between the reassembled soil microbiota by different root exudates and pathogen abundance. We then screened the potential compounds responsible for reducing pathogen abundance and evaluated the concentrations of key compounds in root exudates and their effects on pathogenic strains. This study provides insight into how the root exudates of plants grafted onto resistant rootstock contribute to reducing crown gall disease.

**Figure 2:**
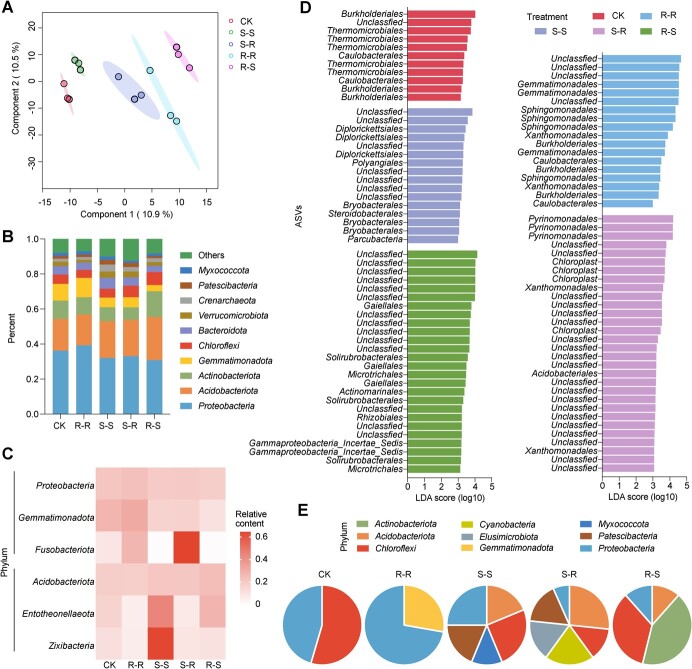
Composition of bacterial communities in soil. **A** PLS-DA of the bacterial community in soil with root exudates from different types of grafted plants. **B** Relative abundance of the top 10 phyla of the soil bacterial community in different treatments. **C** Relative content of phyla in the bacterial community in different treatments. **D** Indicator ASVs in soil treated with root exudates of different grafted plants. ASVs at order level with linear discriminant analysis (LDA) scores >3.0 were analyzed by LEfSe. **E** The pie plots show the percentage of indicator ASVs from different treatments at the phylum level. Sterile water was used as a control.

**Figure 3:**
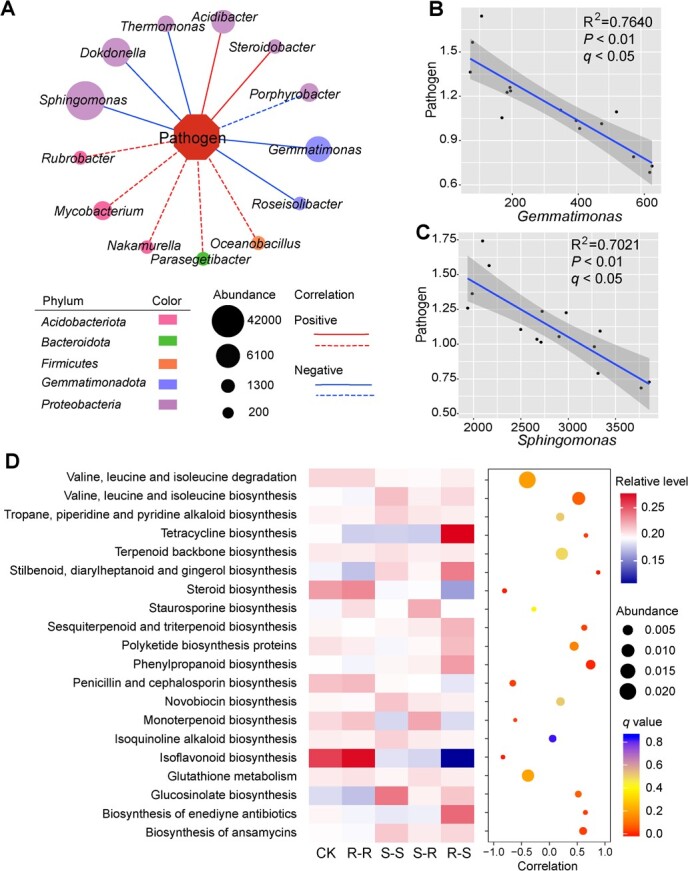
Correlation of the abundance of pathogenic *Agrobacterium* with genera, beneficial bacteria, and bacterial functions. **A** Correlation of pathogen abundance with identified bacteria at genus level (*P* < 0.01). Solid lines indicate significant correlation at *q* value <0.05. Dashed lines indicate *P* < 0.01. Different colors of nodes indicate the phylum of bacteria. Relationships of pathogen abundance with two potential beneficial bacterial groups, *Gemmatimonas* (**B**) and *Sphingomonas* (**C**). **D** Analysis of KEGG pathways related to disease defense in different treatments. The color scale of the heat map represents the relative content of the function in different treatments. Pearson correlation between KEGG pathways and abundance of pathogenic *Agrobacterium* is presented in the bubble map. The color scale of the bubble map indicates the *q* value.

## Results

### Resistant cultivar as rootstock reduces the abundance of tumorigenic bacteria in naturally contaminated soil

The cherry cultivars ‘Haiying 1’ and ‘Gisela 6’ are generally used rootstocks for sweet cherry grafting. Cultivar ‘Haiying 1’ is resistant to crown gall disease (R), while ‘Gisela 6’ is susceptible to this disease (S) in the natural condition (Supplementary Data Fig. S1A and B). However, they showed no significant difference in disease incidence and index against tumorigenic strains directly in the sterile condition (Supplementary Data Fig. S1C, D). This indicated that the soil microbiome is an important factor contributing to the crown gall disease resistance of ‘Haiying 1’ seedlings. One-year-old seedlings of the resistant cultivar and susceptible cultivar were collected for grafting, including grafting the resistant cultivar to the susceptible cultivar rootstock (R–S), grafting the susceptible cultivar to the resistant cultivar rootstock (S–R), grafting the resistant cultivar to the resistant cultivar rootstock (R–R) and grafting the susceptible cultivar to the susceptible cultivar rootstock (S–S). The crown gall disease index of the grafted plants was tested. R–R plants have the lowest disease index, while S–S plants and R–S plants have a much higher disease index. S–R showed only half of the disease index of S–S and R–S plants (Fig. 1A). The rhizosphere bacterial community of different grafted plants was analyzed (Supplementary Data Table S1). The six most abundant phyla were Proteobacteria, Acidobacteriota, Actinobacteriota, Gemmatimonadota, Chloroflexi, and Bacteroidota, accounting for 83.06–86.32% of the total observed amplicon sequence variants (ASVs) (Supplementary Data Fig. S2A). Partial least squares discriminant analysis (PLS-DA) showed that the bacterial communities assembled in different grafted plants rhizosphere were distinct (analysis of similarity (ANOSIM) *R* = 0.9393, *P* = 0.001) (Supplementary Data Fig. S2B).

Previous studies have shown that root exudates play key roles in shaping the composition of the soil microbiome [14]. We wondered whether root exudates from grafted plants with resistant rootstocks would reduce the abundance of tumorigenic strains in naturally contaminated soil. We identified an organism that belonged to the *Agrobacterium* genus and was most closely related to *Agrobacterium tumefaciens* (99.41%). The root exudates were collected from four treatments, R–S, S–R, R–R, and S–S. The amount of carbon or the ratio of carbon/nitrogen added to the soil from different root exudates was calculated and no significant difference was observed (Supplementary Data Fig. S3). We treated the same homogenized soil with root exudates collected from different grafted plants and measured the abundance of pathogenic *Agrobacterium* in the soil. As expected, treating soil with root exudates from R–R decreased the abundance of pathogenic *Agrobacterium* in comparison with untreated soil, while treating soil with root exudates from S–S increased that abundance (Fig. 1B). Interestingly, the abundance of pathogenic *Agrobacterium* in soil treated with root exudates from S–R was lower than the abundance of pathogenic *Agrobacterium* in soil treated with root exudates from S–S treatment. However, root exudates from R–S did not reduce the abundance of pathogenic *Agrobacterium* compared with S–S (Fig. 1B). These results indicated that the root exudates from a grafted plant with resistant rootstock could reduce the abundance of pathogenic *Agrobacterium* in soil.

In addition, the crown gall disease index of the grafted plant showed a profile similar to the profile of the abundance of pathogenic *Agrobacterium* in soil (Fig. 1A). The disease index was positively correlated with the abundance of pathogenic *Agrobacterium* (Fig. 1C). These findings indicated that the reduced abundance of pathogenic *Agrobacterium* in soil affected by root exudation may play an important role in the decreased disease index of the plants grafted onto resistant rootstock.

### Root exudates from different grafted plants reassemble bacterial communities

Root exudates and soil bacterial communities play important roles in plant disease resistance. To understand how the root exudates from grafted plants altered the soil bacterial communities, we analyzed the bacterial communities in soil treated with root exudates from grafted plants by high-throughput sequencing. Soil treated with sterile water was included as a control (CK). In total, 9192 bacterial ASVs were detected and used for further analysis of the bacterial community (Supplementary Data Table S2). Venn analysis showed that the number of ASVs unique to the CK, R–R, S–S, S–R, and R–S treatments was 842, 495, 1006, 976, and 703, respectively (Supplementary Data Fig. S4A). All these unique ASVs accounted for 43.76% of the total observed ASVs. A total of 1533 ASVs were shared among all treatments and accounted for 16.68% of the total observed ASVs (Supplementary Data Fig. S4A). No significant difference was observed for the alpha-diversity of soil bacterial communities between the treatments (Supplementary Data Fig. S4B and C). PLS-DA showed that the microbial community in soil treated with root exudates in the five treatments was clearly distinct (ANOSIM *R* = 0.6222, *P* = 0.001) (Fig. 2A). The pathogen abundance was negatively correlated with principal component 5 (*P* < 0.05) (Supplementary Data Table S3). The six most abundant phyla were* Proteobacteria*, *Acidobacteriota*, *Actinobacteriota*, *Gemmatimonadota*, *Chloroflexi*, and* Bacteroidota*, accounting for 77.94–86.34% of the total observed ASVs (Fig. 2B). At the phylum level, the relative abundances of the three phyla *Proteobacteria*, *Gemmatimonadota*, and* Fusobacteria* in the soil were increased by root exudates from grafted plants with resistant rootstocks (S–R and R–R) compared with grafted plants with susceptible rootstocks (S–S and R–S, respectively), (*P* < 0.05), whereas the three phyla* Acidobacteriota*, *Entotheonellaeota*, and *Zixibacteria* in the soil were reduced (*P* < 0.05) (Fig. 2C). The profile of abundance of ASVs with highest 16S sequence similarity to pathogenic *Agrobacterium* was similar to that of the abundance of pathogenic *Agrobacterium* measured by qPCR (Supplementary Data Fig. S5).

Indicator analysis revealed that root exudates from grafted cherry plants affected the relative abundance mainly of *Proteobacteria*, *Acidobacteriota*, and *Chloroflexi* (Fig. 2D and E and Supplementary Data Table S4). Root exudates of each grafted plant corresponded to a specific subset of phyla. For example, a high proportion of ASVs indicative of root exudate of S–S plants belonged to *Chloroflexi* and *Proteobacteria* (50%), a high proportion of ASVs indicative of root exudate of S–R plants belonged to *Acidobacteriota* and* Cyanobacteria *(46.67%), and a high proportion of ASVs indicative of root exudate of R–S plants belonged to *Actinobacteriota *and *Chloroflexi* (76.92%) (Fig. 2D and E). Eighteen ASVs indicative of root exudate of R–R plants belonged to *Proteobacteria* and *Gemmatimonadota* (100%), and seven ASVs among these ASVs belonged to the *Gemmatimonadales* and *Sphingomonadales *orders, which were putatively plant-beneficial bacterial groups (Fig. 2D and E).

### Correlation of pathogenic *Agrobacterium* with bacterial community and function

To understand the impact of the reassembled bacterial community on pathogen abundance, the correlation of pathogens with other bacteria was analyzed. We found that 13 identified genera were significantly correlated with the abundance of pathogenic *Agrobacterium* (*P* < 0.01), among which 7 genera correlated with the abundance of pathogenic *Agrobacterium* with *q* value (Benjamini–Hochberg-adjusted *P*) <0.05 (Fig. 3A). The abundance of *Sphingomonas* was the highest among the genera. Two genera, *Acidibacter* and *Steroidobacter*, were significantly (*q* < 0.05) and positively correlated with the abundance of pathogenic *Agrobacterium*. The other five genera, including *Sphingomonas*, *Dokdonella*, *Thermomonas*, *Gemmatimonas*, and *Roseisoilibacter*, were significantly (*q* < 0.05) and negatively correlated with pathogen abundance (Fig. 3A). Among these genera, *Gemmatimonas* and *Sphingomonas* were putatively beneficial genera (Fig. 3B and C).

To evaluate how the soil potential function was changed, we predicted the community functions using Tax4Fun software based on the 16S rRNA dataset [33]. The comparison of predicted genes related to disease resistance in the Kyoto Encyclopedia of Genes and Genomes (KEGG) pathways of reassembled soil bacterial communities was analyzed. Compared with S–S plants, several KEGG pathways were enriched by S–R plants, including ‘steroid biosynthesis’, ‘penicillin and cephalosporin biosynthesis’, ‘monoterpenoid biosynthesis’, and ‘isoflavonoid biosynthesis’, which showed a significant negative correlation with pathogenic *Agrobacterium* (*q* < 0.05) (Fig. 3D, Supplementary Data Table S5).

### Correlation of root exudates and pathogenic *Agrobacterium* abundance

**Figure 4:**
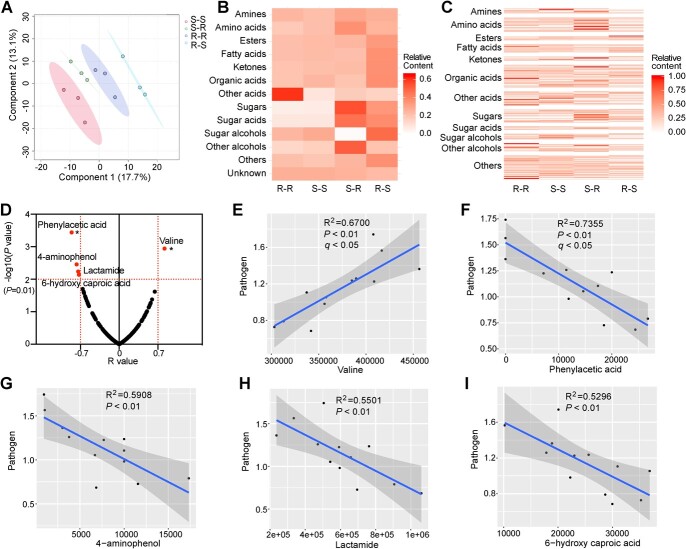
GC–TOF–MS analysis of root exudate composition. **A** PLS-DA score plots of root exudates of different grafted seedlings. **B** Relative content of root exudates of different grafted seedlings in groups. **C** Relative content of compounds in root exudates of different grafted seedlings. **D** Correlation between compounds in root exudates and pathogenic *Agrobacterium* abundance. The horizontal line (y-axis=2) represents a *P* value of 0.01. The vertical lines (x-axis=-0.7 and 0.7) represent *R* values of −0.7 and 0.7, respectively. The points above the horizontal line (y-axis=2) indicate that the *P* value of the correlation between these identified compounds and pathogen abundance was <0.01. ^*^q value <0.05. **E**–**I** Correlations of abundances of valine, phenylacetic acid, 4-aminophenol, lactamide, and 6-hydroxy caproic acid with pathogenic *Agrobacterium* abundance.

To identify the compounds in root exudates that are responsible for regulating the abundance of pathogenic *Agrobacterium* in soil, the composition of root exudates was analyzed. The identified compounds were classified into 12 groups based on their specific structures (Supplementary Data Table S6). We identified 262 metabolic features, including 8 amines, 23 amino acids, 7 esters, 13 fatty acids, 7 ketones, 36 organic acids, 22 other acids, 21 sugars, 5 sugar acids, 9 sugar alcohols, 13 other alcohols, and 36 others. PLS-DA showed that the root exudate profiles of the four treatments were clearly separated from each other (Fig. 4A). The pathogen abundance was negatively correlated with principal component 3 of root exudates (Supplementary Data Table S7). The relative content of root exudates and classes under the different treatments is presented in the heat maps in Fig. 4B and C.

**Figure 5:**
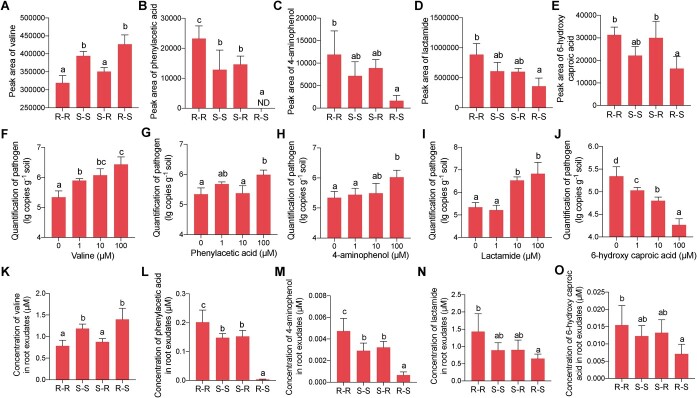
Content of potential signals in root exudates of different grafted plants and the impact of these potential signals on pathogenic *Agrobacterium* abundance. **A**–**E** Peak areas of valine (**A**), phenylacetic acid (**B**), 4-aminophenol (**C**), lactamide, (**D**) and 6-hydroxy caproic acid (**E**) analyzed by GC–TOF–MS. **F**–**K** Quantification of pathogenic *Agrobacterium* in soil treated with concentrations from 0 to 100 μM valine (**F**), phenylacetic acid (**G**), 4-aminophenol (**H**), lactamide (**I**), and 6-hydroxy caproic acid (**J**). **K**–**O** Concentrations of valine (**K**), phenylacetic acid (**L**), 4-aminophenol (**M**), lactamide (**N**), and 6-hydroxy caproic acid (**O**) in root exudates of different grafted plants were analyzed by UHPLC. Values are mean ± standard deviation of three replicates. Different letters indicate statistically significant differences between treatments (*P* < 0.05).

To further explore the potential signals, a Pearson correlation analysis of abundance between pathogenic *Agrobacterium* and each compound in root exudates was performed. Five identified compounds with the highest confidence (*P* < 0.01), including valine (*q* < 0.05), phenylacetic acid (*q* < 0.05), 4-aminophenol, lactamide, and 6-hydroxy caproic acid, were selected as potential compounds responsible for affecting the abundance of pathogens (Fig. 4D). The abundance of valine was positively correlated with the abundance of pathogenic *Agrobacterium* (Figs 4E and 5A). The abundances of phenylacetic acid, 4-aminophenol, lactamide, and 6-hydroxy caproic acid negatively correlated with the abundance of pathogenic *Agrobacterium* (Figs 4F–I and 5B–E). In addition to pathogenic *Agrobacterium*, many other ASVs belonging to the Actinobacteriota and Proteobacteria were significantly correlated with these five compounds (Supplementary Data Fig. S6).

### Effect of potential signals in root exudates on soil pathogenic *Agrobacterium*

We wondered whether these five compounds have a direct effect on pathogenic *Agrobacterium* abundance. We applied the five pure compounds to the soil and determined the abundance of pathogenic *A. tumefaciens* in diseased soil. The abundance of pathogenic *Agrobacterium* in soil increased with increasing valine, 4-aminophenol, or lactamide (Fig. 5F, H, and I) and decreased with increasing 6-hydroxy caproic acid content (Fig. 5J). No obvious trend was observed between the pathogen and phenylacetic acid (Fig. 5G).

To further confirm whether these compounds were present in root exudates at effective concentrations, we quantitatively measured the concentrations of valine, 4-aminophenol, 6-hydroxy caproic acid, phenylacetic acid, and lactamide by UHPLC–MS based on standards (Supplementary Data Fig. S7). The results showed that phenylacetic acid, 4-aminophenol, and 6-hydroxy caproic acid were all present in root exudates in trace amounts (Fig. 5L, M, and O). Although the concentration of lactamide was 1 μM, lactamide did not show a significant effect on the abundance of *A. tumefaciens* (Fig. 5I and N). The reduction of valine concentrations in root exudates has been demonstrated to have an impact on *A. tumefaciens*. We also found that the concentration of valine in root exudates of S–R was reduced compared with the concentration of valine in root exudates of S–S (Fig. 5A and K). In conclusion, we speculated that reduced valine in root exudates of plants grafted on the resistant cultivar rather than on the susceptible cultivar contributes to the reduced abundance of *A. tumefaciens*, consistent with the correlation analysis.

### Relationship of valine with pathogen and bacterial community

**Figure 6:**
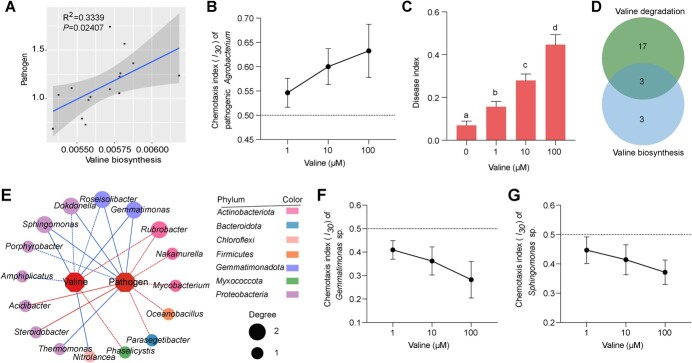
Analysis of the effect of valine on the pathogen and bacterial community. The relationship of the valine biosynthesis pathway (**A**) with pathogenic *Agrobacterium* abundance is presented. The chemotaxis index (*I*_30_) of pathogenic *Agrobacterium* (**B**) and disease index of ‘Haiying 1’ cherry plant (**C**) with different concentration of valine were tested. An *I*_30_ value >0.55 indicates strains are attracted by the compound. An *I*_30_ value between 0.45 and 0.55 indicates that strains have no response to the compound. An *I*_30_ value <0.45 indicates that strains are repelled by the compound. **D** Venn diagram of bacteria at the genus level significantly correlated with the valine biosynthesis pathway or the valine degradation pathway (*P* < 0.01). **E** Correlation of bacteria at the genus level with valine content or pathogen abundance (*P* < 0.01). Solid lines indicate a significant difference at *q* value <0.05. Dashed lines indicate *P* < 0.01. The chemotaxis of *Gemmatimonas* sp. (**F**) and *Sphingomonas* sp. (**G**) was tested using the SlipChip device.

To further analyze the relationship between valine and pathogenic *Agrobacterium*, we predicted valine-related functions in soil and tested the direct impact of valine on pathogen chemotaxis and crown gall disease. We found that the predicted functional level of valine synthesis was positively correlated with the abundance of the pathogen (*P* < 0.05) (Fig. 6A), consistent with the direct effect of valine on pathogen abundance (Fig. 5F). In addition, with increasing concentration of valine, the chemotaxis of pathogenic *Agrobacterium* was enhanced (Fig. 6B). The disease index also increased with increasing concentration of valine (Fig. 6C). These results revealed that the reduced valine in root exudates of plants grafted on the resistant cultivar may contribute to the suppression of crown gall disease.

Since both reduced root exudation of valine and the enriched beneficial microbes in the rhizosphere community were supposed to play a role in reducing the abundance of *A. tumefaciens*, we wondered whether the rhizosphere bacterial community functions are associated with valine. Venn analysis showed that the number of genera that significantly correlated with pathways unique to valine biosynthesis and valine degradation was 3 and 17, respectively (*P* < 0.01). The number of genera that significantly correlated with both pathways was 3 (*P* < 0.01). Most genera were significantly correlated with valine degradation (Fig. 6D). Among these genera, the abundance of *Gemmatimonas* was significantly and positively correlated with the valine degradation pathway (*q* value <0.05) and negatively correlated with the valine biosynthesis pathway (*P* < 0.01) (Supplementary Data Fig. S8).

Five genera were significantly correlated with both valine and pathogen abundance, and belonged to the Proteobacteria, Actinobacteria, and Gemmatimonadota phyla. Among these genera, the putatively beneficial genera *Gemmatimonas* and *Sphingomonas* were negatively correlated with both valine and pathogen abundance (*q* value <0.05) (Fig. 6E). Based on these results, we speculated that the decreased valine exudation by roots and the altered microbiome composition are associated. We measured the direct effect of valine on the beneficial bacteria *Gemmatimonas* sp. and *Sphingomonas* sp. The result showed that valine, at final concentrations of 1, 10, and 100 μM, acted as a repellant of *Gemmatimonas* sp. and *Sphingomonas* sp. (Fig. 6F and G). Moreover, the growth of *Gemmatimonas* sp. and *Sphingomonas* sp. was significantly reduced by 100 μM (Supplementary Data Fig. S9A and B). In conclusion, the effects of valine on chemotaxis and growth of *Gemmatimonas* sp. and *Sphingomonas* sp. were consistent with the correlation analysis (Fig. 6E). These results indicated that the reduced valine in root exudates of plants grafted on the resistant cultivar contributed to enriching the beneficial bacteria.

## Discussion

Crown gall disease is a widespread soil-borne disease. Grafting plants onto resistant rootstocks has been proven to be an effective method to assist plants resist soil-borne diseases [34]. In this study, we explored the impact of root exudates of different grafted plants on disease suppression, soil bacterial community and function. Root exudates altered by the interplay between scion and rootstock would change the composition of the bacterial community and then indirectly affect pathogen abundance. In addition, some compounds in root exudates contribute directly to the reduction of pathogen abundance and improve plant health (Supplementary Data Fig. S10). Therefore, our objective was to assess the importance of the change in root exudates caused by grafting in altering the soil bacterial community and improving the disease resistance of grafted plants.

Previous studies have demonstrated that transmissible signals between scion and rootstock, including RNAs, proteins, and hormones, contribute to enhancing plant resistance against soil-borne crown gall disease through grafting onto resistant rootstocks [35–37]. In addition to these mechanisms inside the grafted plants that would improve their systemic resistance, the role of root exudates in the rhizosphere in helping grafted plants resist disease is still unclear. Root exudates in natural soil conditions are hard to collect due to the contamination by soil organic compounds [14, 38]. It should be noticed that root exudates collected from a hydroponic environment are different from those collected from the natural soil environment, but can reflect plant responses. In this study, we found that changes in both the rootstock and scion have a significant impact on the composition of root exudates, but for soil-borne disease, resistant plants used as rootstocks but not scions can improve susceptible plant disease resistance. We speculated that root exudates are more affected by rootstocks than scions and that the alteration of some compounds in root exudates by grafting onto resistant rootstocks can reduce pathogen abundance in soil to improve the soil environment and maintain plant health (Supplementary Data Fig. S10).

We found that the decrease in valine contributes to the suppression of crown gall disease in grafted plants with resistant cherry rootstock. Valine could block the *Agrobacterium* transportation of γ-aminobutyric acid (GABA), which functions in inducing the degradation of the quorum sensing signal that triggers the infectivity of the *Agrobacterium*, thus favoring crown gall disease [39, 40]. Our previous study showed that valine in root exudates is an important signal that induces chemotaxis, biofilm formation, and plant colonization with *A. tumefaciens* C58 [41]. In that study, we found that plants reduced root secretion of valine upon pathogen attack to defend themselves. Here, we demonstrated that roots of crown gall disease-resistant cultivars secreted less valine than the susceptible cultivar roots, while grafting onto a cultivar secreting less valine helped to protect against crown gall disease.

Root exudates affect plant performance by shaping soil microbiota and play a critical role in plant–soil microbiome interactions [12, 24, 42]. In this study, the soil bacterial composition was significantly altered by root exudates of different grafted plants. Previous studies have demonstrated that the soil microbial composition affects plant disease resistance and that the soil microbiota from resistant plants can reduce disease symptoms in susceptible plants [23]. The negative relationship between some beneficial bacteria and pathogenic *Agrobacterium* would participate in plant disease defense [43, 44]. These bacterial groups include *Sphingomonas* sp. and *Gemmatimonas* sp., which could protect plants from disease [45–48]. These assembled beneficial bacteria might contribute to the reduction in disease severity of grafted plants with resistant rootstocks. Moreover, the abundance of the potential beneficial microbes *Gemmatimonas* and *Sphingomonas* negatively correlated with valine abundance. Some studies have also reported that the abundance of *Sphingomonas* was negatively correlated with valine abundance in ginseng rhizosphere soil or rice seed [49, 50]. The abundance of *Gemmatimonas* was negatively correlated with valine in a blueberry orchard [51]. We speculated that the reduced valine in root exudates and altered composition of the bacterial community may be linked.

In addition to the direct effect of root exudates on pathogenic *Agrobacterium*, this study provides a potential functional link between root exudate-dependent changes in the soil bacterial community and plant disease resistance. We predicted the microbiome function and found that the predicted valine biosynthesis was positively correlated with pathogenic *Agrobacterium* abundance. Besides the KEGG pathways related to valine, several KEGG pathways associated with disease-resistant function were also analyzed. Some pathways were enriched in plants grafted on resistant rootstock (R–R, S–R), including steroid biosynthesis, penicillin and cephalosporin biosynthesis, monoterpenoid biosynthesis, and isoflavonoid biosynthesis. Steroids are related to bacterial and fungal disease resistance [52, 53]. Penicillin and cephalosporin are common antibiotics produced by soil microorganisms [54]. Some volatile compounds belonging to the monoterpenoids are antibacterial compounds that can kill pathogenic *Agrobacterium* [55, 56]. Isoflavones play diverse roles in plant–microbe interactions, especially in plant defense responses [57, 58]. These predicted disease-resistance functions of soil bacterial communities could probably contribute to the reduction of pathogenic *Agrobacterium* abundance.

In conclusion, our results demonstrate that seedlings grafted on resistant rootstock favor reduction of pathogen abundance in soil, and the altered root exudates may play important roles in regulating the soil bacterial community and resisting crown gall disease. Among these altered root exudates, the decreased valine in root exudates of plants grafted on resistant rootstock was thought to play a critical role in decreasing pathogenic *Agrobacterium* abundance in soil. Resistant plants used as rootstocks but not scions would be more conducive to resisting crown gall disease, which is probably caused by regulating root exudates, governing the assembly of beneficial microorganisms, and changing soil microbiome function. The important aspect of this study is that it provides insights into the mechanism by which grafted plants suppress soil-borne disease.

## Materials and methods

### Plant materials and growth conditions

Crown gall-resistant seedlings of ‘Haiying 1’ (*Prunus pseudocerasus* Lindl) and susceptible seedlings of ‘Gisela 6’ (*Prunus cerasus× P. canescens*), obtained from the Laboratory of Plant Tissue Culture Technology of Haidian District, Beijing, China, were cultivated in a greenhouse with a 14/10 h day/night photoperiod at temperatures ranging between 25 and 30°C.

Grafted R–S seedlings were obtained by grafting 1-year-old ‘Haiying 1’ (resistant, R) onto 1-year-old ‘Gisela 6’ (susceptible, S) rootstocks. Grafted S–R seedlings were obtained by grafting ‘Gisela 6’ onto ‘Haiying 1’ rootstocks. R–R refers to self-grafted ‘Haiying 1’ seedlings. S–S indicates self-grafted ‘Gisela 6’ seedlings. To ensure successful grafting, the grafting site was sealed with film, and the grafted seedling was covered with plastic film for 10 days. Then, seedlings were grown in pots and acclimated in the natural conditions of a greenhouse with the same light and temperature conditions.

### Tumor formation in different plants

One-year-old ungrafted seedlings (‘Haiying 1’ and ‘Gisela 6’) and grafted seedlings at the six- to seven-leaf stage were used for inoculation with pathogenic *Agrobacterium*. The roots of these seedlings were wounded at three sites with sterile needles and infected with pathogenic *A. tumefaciens* isolated from the sampling field at a density of 5 × 10^6^ CFU g^−1^ soil as described by Chen *et al*. [41]. In addition, surface-sterilized seedlings were directly infected with pathogenic *Agrobacterium* at a concentration of 5 × 10^8^ CFU ml^−1^ and cultivated in sterile bottles containing sterilized soil. The seedlings were cultured in a greenhouse with a 14/10 h day/night photoperiod, maintaining temperatures between 25 and 30°C. The severity of crown gall disease was recorded at 60 days after inoculation. The crown gall disease incidence of different cultivars was determined as follows: (number of plants with tumors/total number of plants) × 100%. The disease index (DI) of crown gall disease was calculated based on tumor formation and size of tumor [43, 44]. In this study, the DI was scored on a scale of 0–5, as described by Chen *et al*. [41]: DI = ∑(number of diseased plants in the index × given disease index)/(total number of plants treated × highest disease index) [59].

### Root exudate collection

The seedlings used for collecting root exudates were from the same batch as plants used for tumor formation. The roots of four types of seedlings (R–R, S–S, R–S, S–R) at the six- to seven-leaf stage were surface-sterilized and rinsed with sterile water. Subsequently, the seedlings were transplanted into 500-ml flasks containing 300 ml of sterile 1/4 sucrose-free Murashige–Skoog (MS) liquid medium, with the seedling roots submerged in the medium. The seedlings were cultured in these flasks for 7 days to allow them to acclimate in hydroponic culture and for root recovery. The MS medium was replaced every 2 days for oxygenation and nutrient addition.

To collect root exudates, the MS medium in flasks was replaced by sterile water. In the next 3 days, the sterile water in the flasks was replaced daily, and the water containing root exudates was collected. The water in the flasks was replaced by MS medium for 2 days to replenish nutrients to the plants. Then, the above steps of root exudate collection were repeated. Each type of seedling was represented by nine samples. Root exudate solutions were filtered through a 0.22-μm membrane (Millipore, Burlington, MA, USA) and then lyophilized. The roots immersed in the water were dried for weighing.

### Root exudate analysis

The root exudates were analyzed by a GC–TOF–MS system at Shanghai Biotree Biotechnology Limited Company, Shanghai, China. The GC–TOF–MS system consisted of an Agilent 7890 gas chromatograph coupled to a time-of-flight mass spectrometer. A DB-5MS capillary column (30 m × 250 μm × 0.25 μm, J&W Scientific, Folsom, CA, USA) was used for this system. The methods of extraction of root exudates and GC–TOF–MS analysis have been described in detail in a previous study [41]. The peaks in QC samples with detection rate <50% or relative standard deviation >30% were removed [60]. The data analysis was performed using the R (v3.6.1) program and MetaboAnalyst (v5.0). The carbon and nitrogen contents in root exudates were measured with an elemental analyzer (vario PYRO cube, Elementar). The combustion tube temperature of the instrument was 850°C and the reduction tube temperature was 650°C.

### Soil treatment and sampling

The soil was collected from cherry orchards with crown gall disease in Haidian District, Beijing, China. Fresh soil samples were obtained from the upper 20 cm of the soil, sieved with a 2-mm sieve and homogenized. Samples for pot experiments were stored at room temperature (25°C) until use. On the one hand, 100 ml of the original concentration of each type of root exudate was added to each pot containing 200 g of soil every week. The root exudates were added to the soil continuously for 6 weeks with a 14/10 h day/night photoperiod, maintaining temperatures between 25 and 30°C, and then the soil was sampled.

On the other hand, different types of grafted seedlings were transplanted into each pot with the same soil contaminated with pathogenic *Agrobacterium*. Then, they were cultured or 6 weeks with a 14/10 h day/night photoperiod and maintained at temperatures between 25 and 30°C. The rhizosphere soil of each seedling was sampled for further analysis, while the soil without seedlings served as the control. Each sample had three replicates.

### DNA extraction and 16S rRNA gene amplicon sequencing

Total DNA of the rhizosphere soil and the soil treated with root exudates was extracted from 0.25 g of each soil sample using a MoBio PowerSoil DNA Isolation Kit (Cat. No. 47014, Qiagen, Shanghai, China). DNA quality was assessed using a NanoDrop spectrophotometer (ND2000, Thermo Scientific, Wilmington, DE, USA). The extracted DNA was stored at −20°C until analysis.

The V3–V4 region of the bacterial 16S ribosomal RNA (rRNA) gene was amplified using the primers 341F and 806R to evaluate the bacterial community. Sequencing was performed using the Illumina NovaSeq 6000 (250 bp paired-end reads; Illumina, San Diego, CA, USA) at Novogene Corporation (Beijing, China). Fast Length Adjustment of Short reads (FLASH) (v1.2.11) was used to merge paired-end reads assigned to samples [61]. High-quality clean tags were obtained by fastp (v0.23.1). The tags were aligned to the SILVA database (v138.1) using the UCHIME (v11) algorithm [62]. The initial ASVs were generated by the DADA2 (v.2020.6.0) module. Taxonomic information annotation was processed using QIIME2 software (v.2020.6) and the SILVA database (v138.1) [63]. The functions of the bacterial community were predicted by Tax4Fun software (v0.3.1). Data analysis was performed by the R (v3.6.1) program and MetaboAnalyst (v5.0).

### Quantification of tumorigenic strains

The abundance of tumorigenic strains (pathogenic *Agrobacterium*) was determined by qPCR analysis using TB Green Premix EX Taq (Takara, San Jose, CA, USA) on an ABI Quantstudio 3D Digital PCR system (Life Technologies, Carlsbad, CA, USA). The specific primers used for the quantification of pathogenic *Agrobacterium* were selected according to previous studies and are specific for virulence region [41]. Copy numbers were calculated based on the values of cycle threshold corresponding to the standard curve.

### Correlation analysis

Pearson correlation was performed using the Hmisc package in R (v.3.6.1) software to examine the relationships between copy numbers of tumorigenic strains, abundance of bacterial genera, soil predicted functional levels, and relative content of each chemical compound in the root exudates of each type of seedling. Correlations with *P* value <0.01 or Benjamini–Hochberg-adjusted *P* (*q* value) < 0.05 were considered as significantly correlated and used for further analysis.

### Quantification of compounds in root exudates

Ten milligrams of freeze-dried root exudate was mixed with 500 μl of extracting solution (acetonitrile:methanol:water = 2:2:1). The samples were centrifuged at 12 000 rpm for 15 min at 4°C. When samples were used for extracting lactamide, the compound still needed to be derivatized with dansulfonyl chloride and saturated sodium carbonate solution in a water bath at 60°C for 30 min. The samples were analyzed by UHPLC–MS using an Agilent 1290 Infinity II Series (Agilent Technologies, Santa Clara, CA, USA) at Wuhan Passion Technology Limited Company, Wuhan, China. The flow rate was 300 μl/min. The mobile phases and elution conditions are shown in Supplementary Data Table S8.

### Chemotaxis assay

The chemotaxis assay was performed using a microfluidic SlipChip device following the method described by Shen *et al*. [64]. A clean SlipChip was assembled under a stereomicroscope. PBS buffer (control), bacterial cells suspended in PBS (pathogenic *Agrobacterium*, *Gemmatimonas aurantiaca*, or *Sphingomonas sanguinis*) and valine at different concentrations (1, 10, and 100 μM), were injected into the top, middle, and bottom microwells, respectively. The SlipChip device was placed in a dark environment for 30 min and then transferred to an inverted fluorescence microscope (Ti-Eclipse, Nikon, Japan). The bacterial cells in the top and bottom microwells were monitored. The chemotaxis index (*I*_30_) was used to indicate the chemotaxis of the bacterial cells with valine at a certain concentration. *I*_30_ is defined as *N*_e_/(*N*_e_ + *N_c_*), where *N*_c_ is the number of bacterial cells that migrated into the PBS-containing microwells and *N*_e_ is the number of bacterial cells that migrated into the valine-containing microwells.

### Statistical analysis

Duncan’s multiple range tests and analysis of variance (SPSS v.25.0) were used to calculate and statistically analyze the differences between the treatments. The *P* value was adjusted by the Benjamini–Hochberg procedure. Student’s *t*-test was used to estimate the significance of paired comparisons. The differentially enriched bacterial taxa in different treatments were identified via linear discriminant analysis effect size (LEfSe, v1.0) analysis. The graphs were generated using the ggplot 2 package in R (v.3.6.1), Prism (v.8.0), Cytoscape (v.3.9.0), and Illustrator 2019.

## Acknowledgements

The authors thank Lanying Liu and Jing Xu at the Laboratory of Plant Tissue Culture Technology of Haidian District for providing ‘Haiying 1’ seedlings. We thank Dr Dongwei Chen and Prof. Wenbin Du (Chinese Academy of Sciences) for help in the chemotaxis assay. This work was supported by the National Natural Science Foundation of China (grants 32370135 and 31700548) and the Innovation Program of Chinese Academy of Agricultural Sciences (CAAS-CSAL-202302).

## Author contributions

Y.L. and L.C. conceived the ideas and designed the methodology; L.B., Y.L., and Q.M. collected the data; L.C. and X.W. analyzed data; L.C. and Y.L. led the writing of the manuscript. All authors contributed critically to the drafts and gave final approval for publication.

## Data availability

The 16S rRNA sequence of the tumorigenic strain in sampling area was deposited in GenBank under the accession number OR056166. The Novaseq paired-end reads for the bacterial 16S rRNA gene V3–V4 region from the rhizosphere soil of grafted seedlings and the soil treated with root exudates have been deposited in the NCBI Sequence Read Archive (SRA) database under the accession numbers PRJNA1073026 and PRJNA896915, respectively.

## Conflict of interest

The authors declare that they have no conflict of interest.

## Supplementary data

Supplementary data are available at *Horticulture Research* online.

## Supplementary Material

Web_Material_uhae049
