# Supplementary material for: Defensive alteration of root exudate composition by grafting Prunus sp. onto resistant rootstock contributes to reducing crown gall disease
Source: Hortic Res. 2024 Feb 23;11(4):uhae049. doi: 10.1093/hr/uhae049 (PMC11031412; doi:10.1093/hr/uhae049)

**Defensive alteration of root exudate composition by grafting *Prunus* sp. onto resistant rootstock contributes to reducing crown gall disease**

Lin Chen^1^, Lusen Bian^1^, Qinghua Ma^1^, Ying Li^1^, Xinghong Wang^1^, Yunpeng Liu^2*^

^1^ National Permanent Scientific Research Base for Warm Temperate Zone Forestry of Jiulong Mountain, Experimental Center of Forestry in North China, Chinese Academy of Forestry, Beijing 102300, People’s Republic of China

^2^ ﻿State Key Laboratory of Efficient Utilization of Arid and Semi-arid Arable Land in Northern China (the Institute of Agricultural Resources and Regional Planning, Chinese Academy of Agricultural Sciences, Beijing 100081, China)

**^*^Corresponding author**

Yunpeng Liu. Tel: +86-010-82105087; Fax: 86-010-82108683; E-mail: liuyunpeng@caas.cn

**Fig. S1** The crown gall disease incidence (A, C) and disease index (B, D) of different cherry cultivars were measured after plants were transplanted into natural soil with pathogenic *Agrobacterium* (A, B) or directly infected with pathogenic *Agrobacterium* in sterile condition (C, D). Values are mean ± standard deviation of 3 replicates. Each replicate contained 20 seedlings. “**” indicates *P*<0.01. “N.S.” indicates no significant difference.


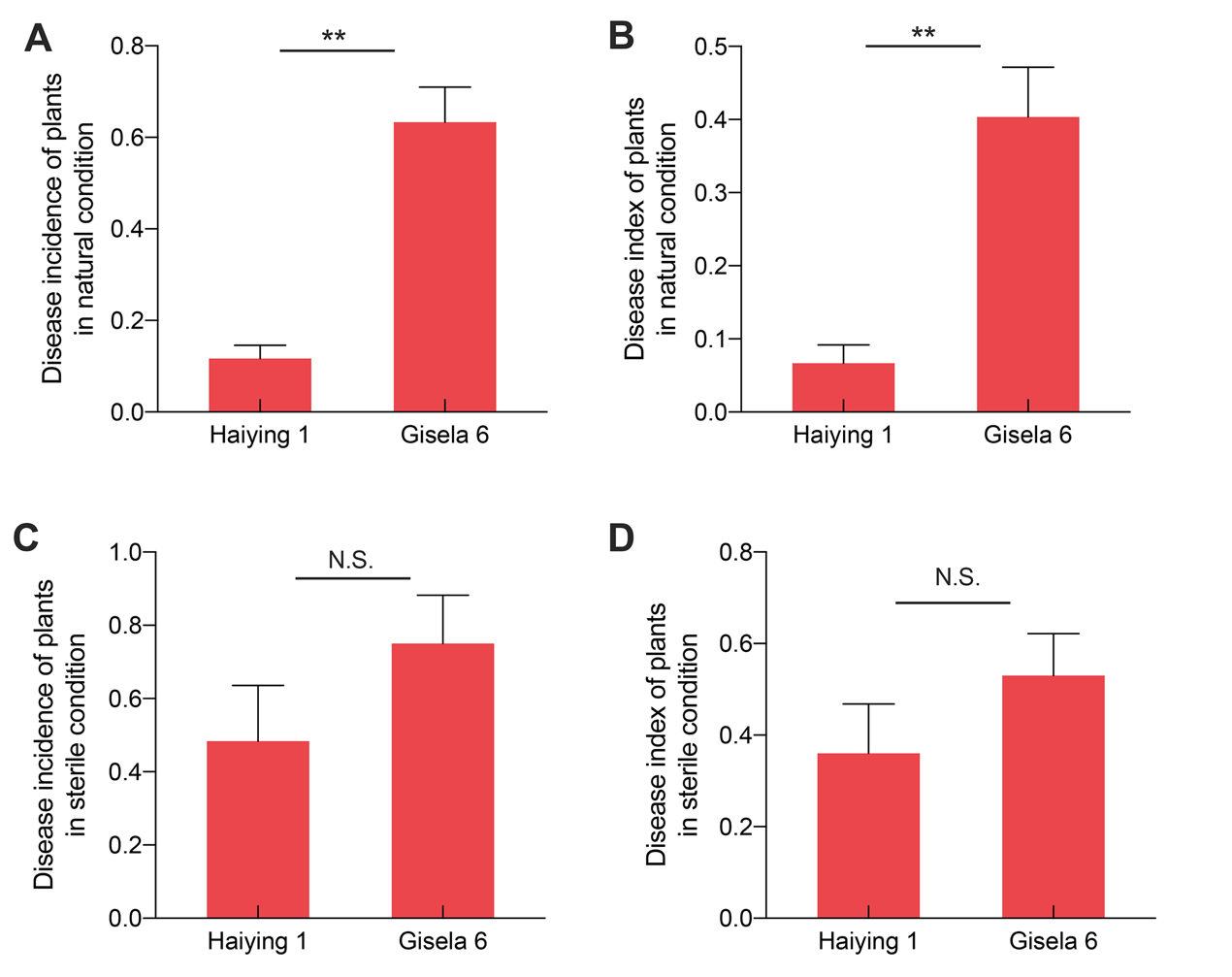


**Fig. S2** Composition of bacterial community in rhizosphere of different grafted plants. (A) Relative abundance of the top 10 phyla of bacterial community in rhizosphere of different grafted plants. (B) Partial least squares-discriminant analysis (PLS-DA) score plots of bacterial community in rhizosphere of different grafted plants. The same soil was planted with different grafted plants for 6 weeks. The control represents soil without plants.


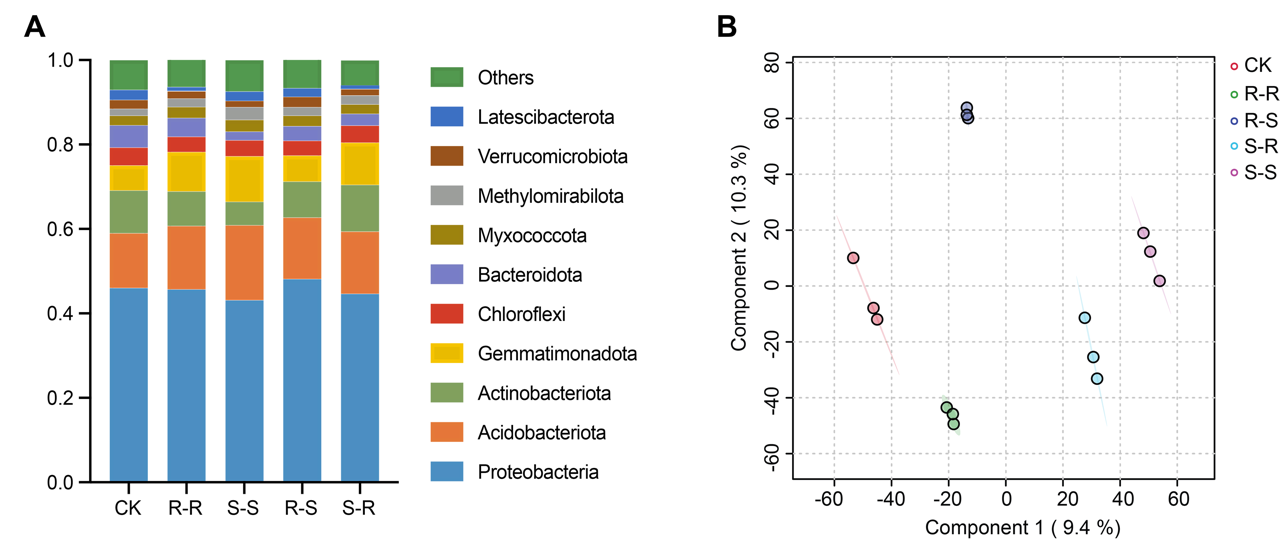


**Fig. S3** The amount of carbon (A) and the ratio of carbon: nitrogen (B) in the root exudates of different grafted plants. The amount of carbon from root exudates added in soil was determined (C). R-R represents grafting the resistant cultivar to the resistant cultivar rootstocks. Abbreviations: S-S represents grafting the susceptible cultivar to the susceptible cultivar rootstocks. S-R represents grafting the susceptible cultivar to the resistant cultivar rootstocks. R-S represents grafting the resistant cultivar to the susceptible cultivar rootstocks. Values are mean ± standard deviation of 3 replicates. “N.S.” indicates no significant difference.


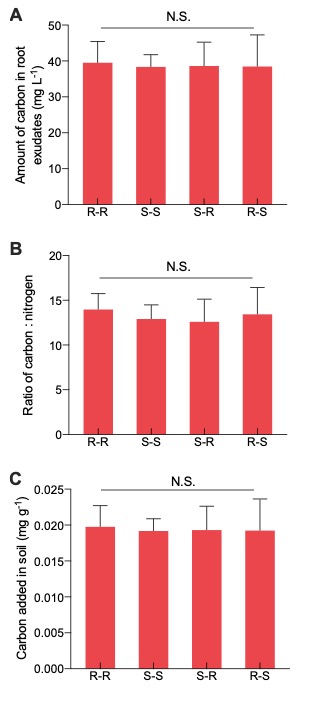


**Fig. S4** Venn diagram (A), Shannon diversity (B) and richness (C) of bacterial communities in soil treated with root exudates of different grafted plants. Sterile water was used as a control. “N.S.” indicates no significant difference.


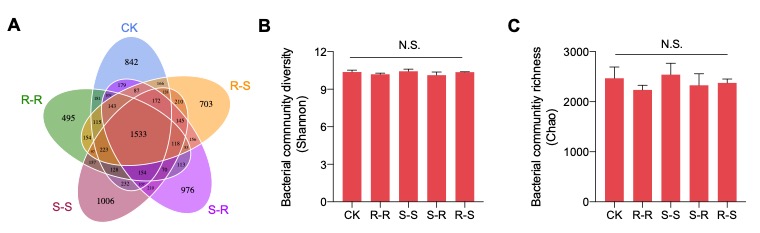


**Fig. S5** Effect of different grafted plants on ASVs with highest 16S sequence similarity to pathogenic *Agrobacterium* (similarity >99%). Values are mean ± standard deviation of 3 replicates. Different letters above the column indicate statistically significant differences between treatments (*P*<0.05).

**Fig. S6** Correlation of five potential signals with bacteria at the genus level. “*” indicates significant difference at *P* < 0.01. “**” indicates significant difference at *q* value < 0.05. The color scale of the heatmap represents the correlation coefficients.

**Fig. S7** UHPLC-MS analysis of compounds in root exudates. The standards and content of valine (A), 4-aminophenol (B), 6-hydroxy caproic acid (C), phenylacetic acid (D) and lactamide (E) in root exudates are presented.


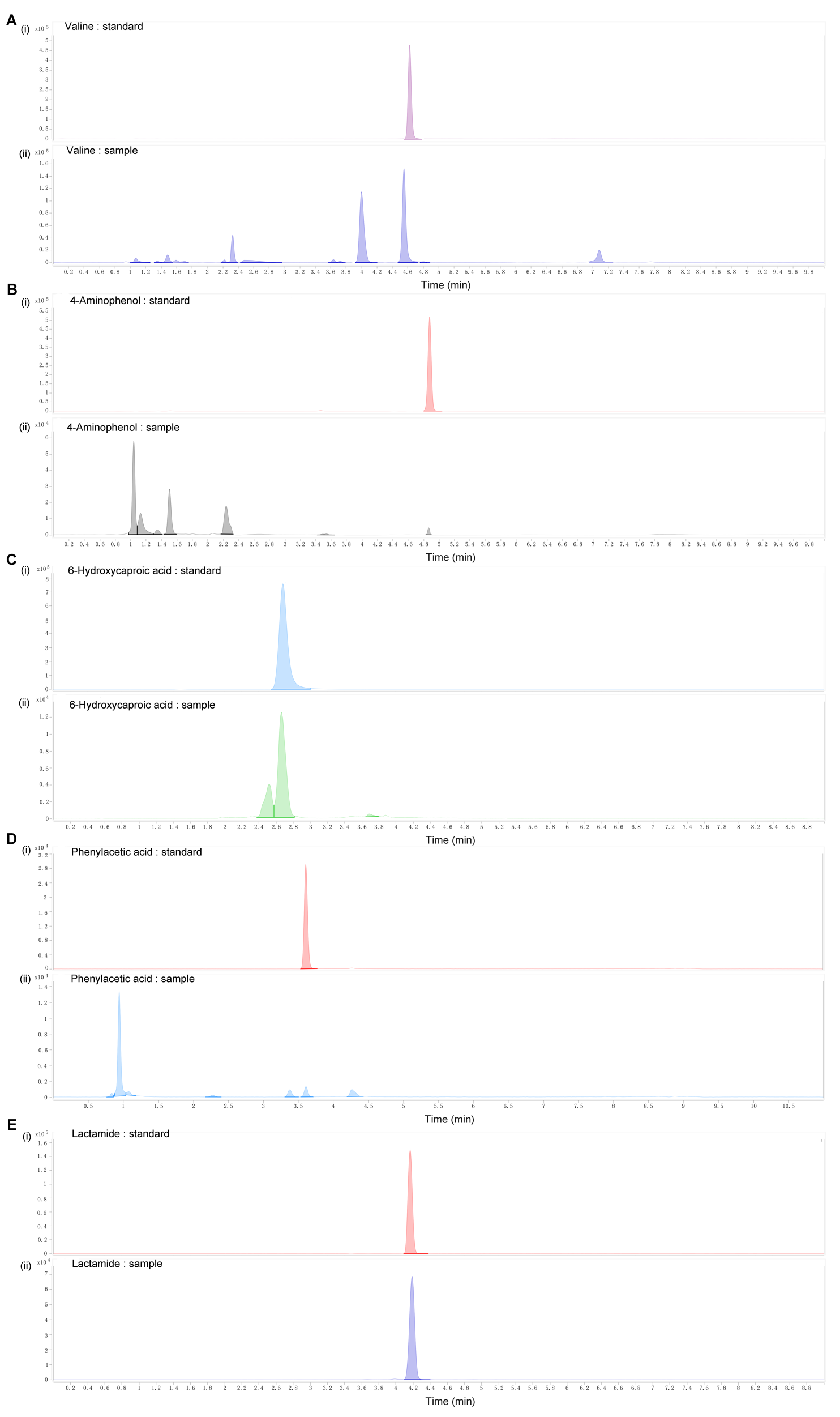


**Fig. S8** Correlation of bacteria at the genus level with valine biosynthesis pathway or degradation pathway. “*” indicates significant difference at *P* < 0.01. “**” indicates significant difference at *q* value < 0.05. The color scale of the heatmap represents the correlation coefficients.

**Fig. S9** Effect of valine on the growth of *Gemmatimonas* sp. and *Sphingomonas* sp. The growth of *Gemmatimonas* sp. in R2A liquid medium (C) and *Sphingomonas* sp. in LB liquid medium (D) with valine was measured at 30 ℃ by an automatic growth curve analyser (Bioscreen C MBR). “*” indicates significant difference between control (0 μM) and valine treatment at *P* < 0.05. The different colors of “*” represent different concentrations of valine treatment.

**
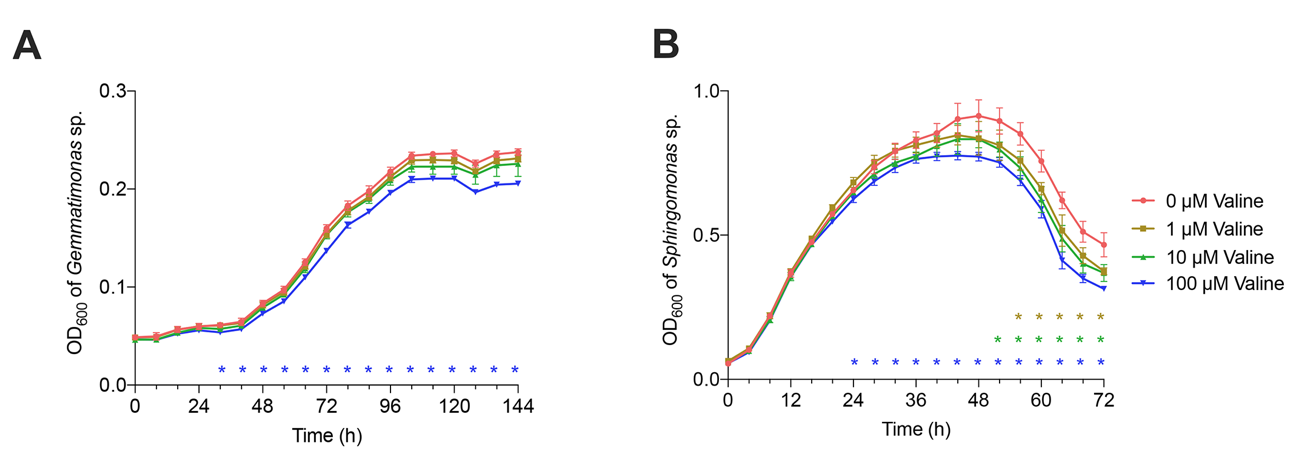
**

**Fig. S10** Model of root exudate function in the resistance of grafted plants with resistant rootstock to crown gall disease. The root exudates were altered by grafting. The root exudates altered by grafting plants onto resistant rootstocks would enrich beneficial bacteria and regulate the function of the reassembled soil bacterial community to inhibit pathogenic *Agrobacterium* indirectly. In addition, some compounds in root exudates also played important roles in directly reducing pathogenic *Agrobacterium* abundance, such as the decrease in valine.


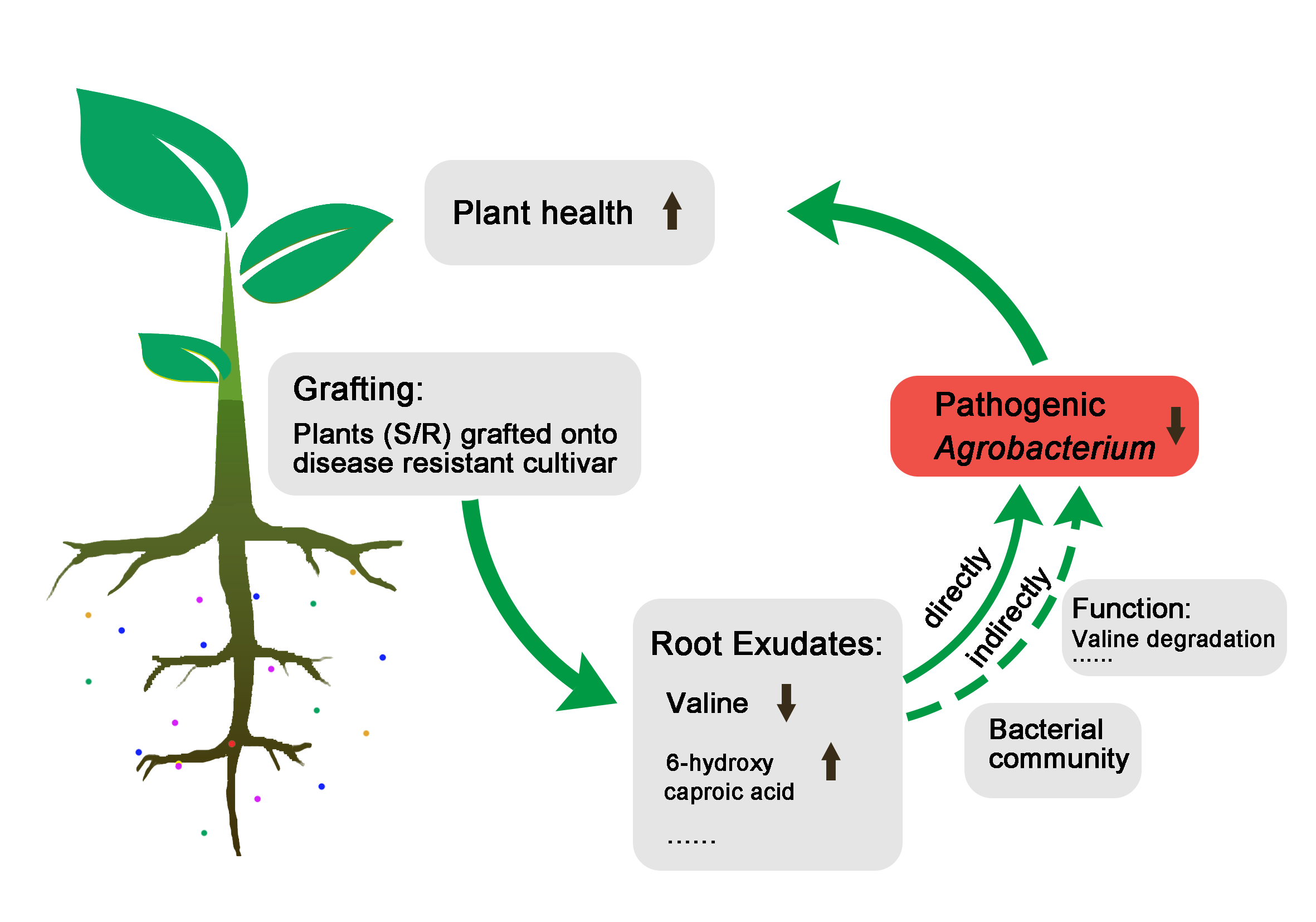

Supplement: Web_Material_uhae049 [file web_material_uhae049.zip › Appendix A -1220-clean.docx]
